# Survival Analysis of Coal Workers’ Pneumoconiosis (CWP) Patients in a State-Owned Mine in the East of China from 1963 to 2014

**DOI:** 10.3390/ijerph14050489

**Published:** 2017-05-06

**Authors:** Lei Han, Qianqian Gao, Jingjin Yang, Qiuyun Wu, Baoli Zhu, Hengdong Zhang, Bangmei Ding, Chunhui Ni

**Affiliations:** 1 Institute of Occupational Disease Prevention, Jiangsu Provincial Center for Disease Control and Prevention, Nanjing 210028, China; hanlei@jscdc.cn (L.H.); gaoshanzhizi@163.com (Q.G.); zhubl@jscdc.cn (B.Z.); hd-zhang@263.net (H.Z.); dingbangmei@163.com (B.D.); 2 Department of Occupational Medicine and Environmental Health, School of Public Health, Nanjing Medical University, Nanjing 210029, China; xjwqy922@163.com (J.Y.); yjjnjmu@163.com (Q.W.); 3 Division of Health Risk Factor Monitoring and Control, Shanghai Municipal Center for Disease Control and Prevention, Shanghai 200336, China

**Keywords:** survival analysis, Coal Workers’ Pneumoconiosis (CWP), China

## Abstract

To investigate the mortality probability, life expectancy of coal workers’ pneumoconiosis (CWP), and related factors of life expectancy, a total of 495 patients with CWP were diagnosed and reported from 1963 to 2014 in a state-owned mine in the east of China. The life table method, log rank method, and Cox regression model were used for survival analysis. 95 out of 495 CWP died during this period. The mortality rate was 19.19%. The average life span was 12.1 (0.0–33.2) years and average death age was 57.4 (33.0–83.0) years. The life table indicated that overall mortality probability increased with the age of CWP patients. Life expectancy of CWP patients was prolonged to 4.3, 1.4, 1.2, and 1.4 years without death caused by pneumoconiosis, tuberculosis, lung cancer, and pulmonary heart disease respectively. The survival curve of CWP patients without pulmonary tuberculosis was higher (average 37.9 years) than patients with pulmonary tuberculosis (average 34.1 years). There was significant difference observed (χ^2^ = 6.196, *p* < 0.05). Three risk factors that include initial dust exposure year, age of onset, and first diagnostic stage were put into the Cox regression model for evaluation. The data indicated that prevention and treatment of CWP complication is important to improve patients’ survival rates.

## 1. Introduction

For a long time, dust has been one of the main occupational hazard factors in China. At present, the mining industry is the most serious dust hazard [1]. There are many types of mining with similar processes, which mainly include exploration, development, operation, decommissioning, and land reclamation. Coal mining is one of the most serious occupational disease hazards in our country. In 2014, the number of occupational disease cases in coal mining accounted for 38.02% of the total number of occupational disease cases reported. The control of dust hazard has become a top priority. However, in coal mines, it is hard to prevent the dust production completely after destruction of a rock mass and coal seam. It is possible to reduce coal mine dust levels for protection miners’ severe Coal Workers’ Pneumoconiosis (CWP), but the resource is expensive and intensive. The most effective measure is decreasing dust concentration in the air of workplaces by adopting the passive preventive measures, such as wet working, timely dust cleaning, reducing wind velocity, and reducing the damage to rock and coal seams during tunneling [2]. Coal mine dust concentration is still relatively high, and the dust is liable to be inhaled by the underground miners, which could lead to lung fibrosis, namely CWP. In addition, the dust could also cause inflammation of the upper respiratory tract, chronic obstructive pulmonary disease (COPD), and so on [3].

CWP refers to a kind of chronic systemic disease characterized by diffuse fibrosis of the lung tissue, which is caused by long-term inhalation and retention of productive dust by coal mine dust exposure workers in occupational activities. Its main pathological changes include coal spot, focal emphysema, coal silicon nodule, diffuse fibrosis, progressive massive fibrosis (PMF), etc. The coal mine workers could respectively be exposed to coal dust, silicon dust, and silicon coal dust due to different types of work in the process of coal mining [4], thus causing coal pneumoconiosis, silicosis, and coal silicosis, collectively known as CWP. There is no specific medicine and effective treatment. Relieving symptoms, reducing complications and slowing disease progression are mainly treatment countermeasures [5,6]. Due to the progression and irreversibility of CWP, the patients lose labor ability gradually, reducing quality of life and shortening life expectancy. Besides, the disease puts physical, mental, and economic burdens on patients and their families, as well as affecting the normal production, survival, and sustainable development of enterprise, causing incalculable economic loss to the society [7].

China is one of the countries most seriously affected by productive dust in the world since it has the largest pneumoconiosis and dust exposed population [8]. The Chinese government is very concerned about its prevention and control, and it has put substantial manpower and material resources into establishing a perfect occupational health management and technical service system. Data from the official website of the Ministry of Health of China showed that 26,873 new cases of pneumoconiosis were diagnosed in 2014, accounting for 89.66% of all reported occupational diseases in China, of which 13,846 (51.52%) were CWP. The number of pneumoconiosis patients accumulatively detected is staggering and likely remains the tip of the iceberg. In addition, new cases of CWP increase quickly every year [9]. A total of 17,023 coal workers from the Kailuan Colliery Group were studied. It suggested that the present and future incidence trends of CWP remain high among coal workers in China [10].

A 23-year follow-up study to evaluate the mortality experience of 8899 working coal miners at 31 U.S. coal mines conducted by National Institute for Occupational Safety and Health (NIOSH) showed that exposure to coal mine dust leads to increased mortality, even in the absence of smoking [11].

The British Pneumoconiosis Field Research (PFR), a prospective exposure-response epidemiological study, was conducted by the Institute of Occupational Medicine, on behalf of the British coal industry, between 1953 and 1991. Supplementary studies and data analysis continue to this day. Key risk estimates from the PFR provide a guide to the most recent and most reliable estimates of dust related risks of substantial pulmonary disease, and to the magnitude of the effects. The most reliable estimates of risks of coal workers’ simple pneumoconiosis (CWSP) and progressive massive fibrosis (PMF) were based on about 50,000 observations of men at risk during 25 years of the research. These findings showed that risk of PMF is positively related to dust exposure, age, stature (body mass index, BMI), the presence of CWSP, and the proportion of carbon in the coal. Risks of an attack of PMF rise from about 0.8% at 1.5 mg·m^−3^ to about 5% at 6 mg·m^−3^. Risks of category II CWSP are higher, rising from about 1.5% at a mean concentration of 1.5 mg·m^−3^ to about 9% at 6 mg·m^−3^. Though there has not been a similar study in China, these studies provide detailed information on the mortality experience of coal miners in other countries, and provide a starting point for understanding PMF effects on Chinese coal miners [12].

The coal production base of China National Coal Group Corp. in Jiangsu has nearly 50 years of mining history. The base experienced the establishment of occupational disease prevention laws and regulations as well as the development of dust-proofing technologies, measures, and equipment from scratch. The replacement of coal mining equipment, the implementation of dust control measures, dust exposure of miners, and incidence of CWP of this base can be a good representative one of Chinese state-owned coal mines. To investigate the mortality probability and life expectancy of CWP and related factors of life expectancy, we investigate 495 cases of CWP patients from 1963 to 2014 in this mining area.

## 2. Materials and Methods

### 2.1. Subjects

We investigated all CWP patients in this mining area who were exposed to dust more than half of their length of service. Besides, patients of the study were collectively diagnosed by an institution with qualification in pneumoconiosis diagnosis in Jiangsu Province, China. The diagnosis was implemented according to the appendix of “silica dust workers medical preventive measures” which was enacted in 1963 to 1986. The diagnosis was made based on “GB 5906-1986 pneumoconiosis X-ray diagnostic criteria and principles of treatment” between 1986 and 1997. From 1998 to 2002, we used “GB 5906-1997 X-ray diagnosis of pneumoconiosis” standard for diagnosis and choose “GBZ 70-2002 diagnostic criteria for pneumoconiosis” diagnosis between 2002 and 2009. A “GBZ 70-2009 diagnostic criteria” has been used for pneumoconiosis diagnosis after 2009. All subjects made agreements with patients before they participated in the study. This research protocol was specifically approved by the Ethics Committee of Nanjing Medical University (Nanjing, China) under the number FWA00001501.

### 2.2. Data Source

The data of CWP patients came from diagnostic information of the staff hospital or epidemic prevention departments in this mining area and the database record information of safety department in four mines. The collection deadline was in October 2014. The collected data included date of birth, type of work, initial date of dust exposure, end date of dust exposure, date of first diagnosis of CWP, diagnosis of the period, year of stage enhancement, complication with tuberculosis, etc. The cause of death information was filled based on a uniform standard. The birth, death date and causes of death were verified one by one. The missing data was checked to ensure the integrity and reliability of the data. The classification of deaths was categorized according to the International Classification of Diseases (ICD-10).

### 2.3. Definition of Death Cause and Censored Data

(1) Death cause: died of CWP or its complications, such as tuberculosis, lung cancer [13,14], pulmonary heart disease, etc.
(2) Censored data: death from other causes, such as cardiovascular accidents, liver failure, accidents, and so on, or still alive at the end of observation.

### 2.4. Statistical Analysis

The data was analyzed by SPSS 16.0 (IBM, Armonk, NY, USA) and grouped by age. The group begins at 25 years old, with 5-year intervals, ending at over 70 years old. The expected number of surviving CWP patients and life expectancy were calculated by the mortality rate, the cumulative survival rate, and the death probability on the basis of the abridged current life table. Survival analysis adopted Kaplan-Meier method and Cox proportional hazards regression model (referred to as Cox model) based on partial maximum likelihood estimation of the forward method (Forward: LR). The regression variable assignment is listed in Table 1.

## 3. Results

In this coal mine, 495 cases of CWP were diagnosed from 1963 to 2014 and 95 of them died. The mortality rate was 19.19%. The average life span was 12.1 (0.0–33.2) years. The average death age was 57.4 (33.0–83.0) years.

### 3.1. Death Probability of CWP Patients in Different Age Groups

The death probability of 495 CWP patients in different age groups calculated based on corrected observed patients. The deceased patients are listed in Table 2.

### 3.2. Life Expectancy of CWP Patients in Different Age Groups

Assuming that there were 100,000 CWP patients of the same generation in the 25~ age group, the expected surviving patients and life expectancy (Table 3) for each age group are calculated based on the death probability. It is indicated that the overall death probability increases with the age of CWP patients. This group of CWP patients is expected to survive 25.6 years, while the death probability was 50.00%, and the median survival age is 70.0 years.

### 3.3. Effects of Several Major Causes of Death on Life Expectancy of CWP Patients

In 95 deceased CWP patients, 31 cases died from pneumoconiosis, accounting for 32.63% of all deaths; 12 died from other tumors, accounting for 12.63%; 11 cases died from pulmonary tuberculosis, lung cancer, pulmonary heart disease, accounting for 11.58%. The impact of each cause of death on the life expectancy of CWP patients is shown in Table 4. When removing death caused by pneumoconiosis, tuberculosis, lung cancer, and pulmonary heart disease, the life expectancy of CWP patients was prolonged by 4.3, 1.4, 1.2, 1.4 years, respectively.

### 3.4. Comparison of Survival Curves between CWP Patients with Pulmonary Tuberculosis and Patients without Pulmonary Tuberculosis

**Figure 1:**
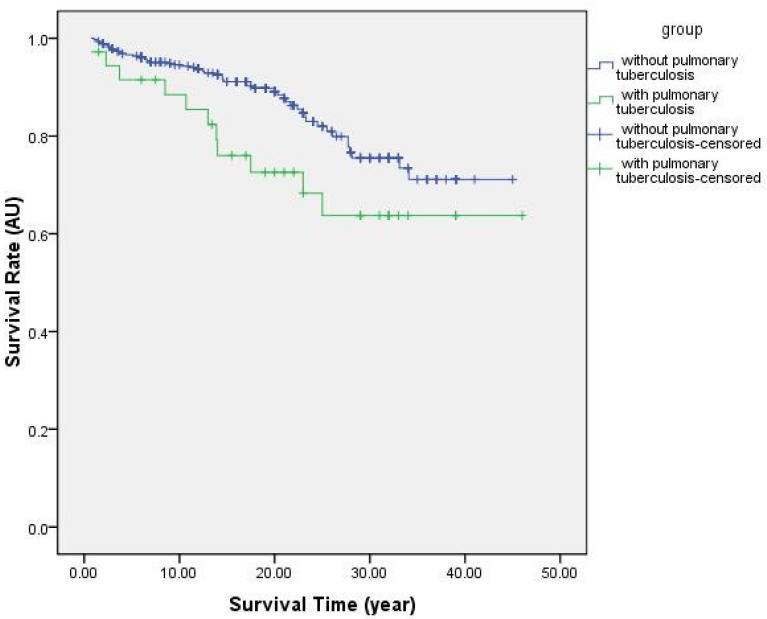
Survival curves of Coal Workers’ Pneumoconiosis (CWP) patients with or without pulmonary tuberculosis.

The Kaplan–Meier method was used to estimate the cumulatively survival rate. 495 CWP patients included 459 cases without pulmonary tuberculosis and 36 cases with pulmonary tuberculosis. 80 out of 459 cases without pulmonary tuberculosis died, the mortality rate was 17.43%, the average dust exposure years was 24.5, the average survival years was 12.4. Of those diagnosed with pulmonary tuberculosis 5 out of 36 patients died, the mortality rate was 41.67%, the average dust exposure years was 24.4, the average survival years of dead patients was 10.8. We found that the survival curve of CWP patients without pulmonary tuberculosis was higher than patients with pulmonary tuberculosis. There is a significant difference observed (χ^2^ = 6.196, *p* < 0.05). The survival curve is shown in Figure 1. The average survival time of surviving CWP patients without pulmonary tuberculosis was 37.9 years, and the average survival time of surviving CWP patients with pulmonary tuberculosis was 34.1 years.

### 3.5. Cox Model Analysis of Factors Influencing Survival Life of CWP Patients

The survival outcome and survival years of CWP were taken as the dependent variables. Initial dust exposure year, dust exposure years, age of onset, time since first exposure, first diagnostic stage, work type, and complication with pulmonary tuberculosis or without were taken as the independent variables. As shown in Table 5, there are three factors, including initial dust exposure year, age of onset, and first diagnostic stage, put into the regression model, which are the risk factors for the survival of CWP patients. The relative risk (RR) of death were 1.897, 0.943, and 0.902 for the first diagnostic stage, age of onset, and initial dust exposure year respectively.

## 4. Discussion

Nowadays, CWP is still believed to be the main occupational disease in China. A systematic analysis of studies from 2001–2011 showed that the pooled prevalence of CWP was 6.02% and the pooled rate of CWP patients combined with tuberculosis was 10.82%. It was concluded that the prevalence of CWP was still high in China compared to UK (0.8%, 1998–2000) and the USA (3.2% in 2000s) [15]. Long-term silica dust exposure was associated with substantially increased mortality among Chinese workers [16]. The increased risk was observed not only for deaths due to respiratory diseases and lung cancer, but also for deaths due to cardiovascular disease.

Between 1964 and 1999, there were 957 cases of CWP patients who died of pneumoconiosis in Xuzhou Mining Group, the average death age of patients without tuberculosis was 65.1 years old [17], slightly higher than 57.4 years old in our survey. The average death age of patients with tuberculosis was 58.9 years old [17]. In the analysis of death causes in CWP patients, mortality rate and life years are indicators of harm severity of CWP. Life table method is a statistical table made based on age-specific mortality rates, which can express the life process of specific population. Besides, it is one of the ideal methods to evaluate the effectiveness of prevention and treatment of chronic diseases. It can also reflect the death and prognosis of CWP patients at different stages after diagnosis more objectively and dynamically. It provides a scientific basis for prolonging age of onset and life years of CWP patients. In this study, we analyzed the life expectancy of 495 diagnosed CWP patients (95 deaths) from a coal mine of east China from 1963 to 2014 using this method. We estimated that the survival years of 100,000 CWP patients of the same generation was 25.6 years, while the cumulatively death probability was 50.0%, and the median survival age was 70.0 years. This is lower than 32.9 years in the investigation of life expectancy of CWP patients in high altitude localities by Shi Chunbo [18]. To estimate the life expectancy, quality-adjusted life expectancy (QALE), and their losses, in patients with pneumoconiosis in Mongolia, a study including 432 pneumoconiosis patients diagnosed during 1986–2006 indicated that the life expectancy and expected years of life loss (EYLL) of a patient with pneumoconiosis were 18.1 and 9.5 years, respectively [19]. In addition, QALE and loss of QALE were 15.1 and 12.5 quality-adjusted life years (QALYs), respectively, indicating a health gap of 45% [19].

To characterize the impact of premature mortality attributed to CWP in the United States, the CDC's National Institute for Occupational Safety and Health (NIOSH) analyzed annual underlying cause of death data from 1968 to 2006 [20]. The results of this analysis indicated that during 1968–2006, a total of 22,625 years of potential life lost (YPLL) were attributed to CWP (mean per decedent: 5.7). Annual YPLL attributed to CWP decreased 91.2%, from an average of 1484 YPLL per year during 1968–1972 to 154 per year during 2002–2006. However, the annual YPLL from CWP has been increasing since 2002, from 135 in that year to 169 YPLL in 2006, suggesting a need for strengthening CWP prevention measures. The CDC intends to maintain surveillance of CWP deaths to determine future trends and promote safer work environments [20].

Tuberculosis is the most common complication of pneumoconiosis patients [21]. Pneumoconiosis with pulmonary tuberculosis is more serious than pneumoconiosis without pulmonary tuberculosis, and it progresses quickly [22]. Pneumoconiosis at different stages with active tuberculosis has been listed into the assessment of occupational injury and occupational disease disability grade. Pneumoconiosis and tuberculosis infection are related to the immune system of the body. Pneumoconiosis can reduce immune function and promote tuberculosis infection or recurrence. Tuberculosis can speed up the disease progression of pneumoconiosis because of the diminished ability of the respiratory tract to remove dust, thus causing a decrease of life span and increased mortality rate. The survey data from Xuzhou Mining Group in the same region showed that 9.69% of CWP patients died of tuberculosis [17], which is similar to our data that CWP patients died of tuberculosis accounted for 11.58% of the total number of deaths. The mean survival time of CWP patients without tuberculosis was 37.9 years, which was higher than 34.1 years of patients with tuberculosis. The data indicated that pulmonary tuberculosis affected the prognosis of CWP patients, and it was one of the main reasons leading to progression of disease and decrease of survival years of CWP patients. In addition, the life expectancy of CWP patients was prolonged by 4.3, 1.4, 1.2, and 1.4 years when removing the death caused by pneumoconiosis, pulmonary tuberculosis, lung cancer, and pulmonary heart disease, respectively. Thus, it is very important to prevent and control complications of pneumoconiosis during its treatments.

Cox model is a multivariate analysis method, which can comprehensively analyze the effects of many prognostic factors, eliminate the interference of confounding factors, and deal with the special censored data of survival data, especially for data of unknown distribution. It is a semi-parametric survival analysis method for death data [18]. There are many factors influencing occurrence of CWP and death of patients [23,24]. The Cox model plays its advantages fully in multivariate analysis when using it to explore related risk factors of survival years in CWP patients [25]. The survey indicates that initial dust exposure year, age of onset, and first diagnostic stage are important risk factors for the CWP patients’ survival. The initial dust exposure year mainly reflects the dustproof condition, and initial dust exposure year ahead indicates increased death risk of CWP patients. Onset of age reflects the individual differences of pneumoconiosis occurrence. The first diagnostic stage hints the severity of disease.

A cohort study including 1847 patients with CWP and 43,742 coal workers without CWP who were registered in the employment records of the Datong Coal Mine Group suggested that, if advanced dustproof equipment was adopted, losses of 1.207 billion RMB would be prevented and 4698.8 healthy life years would be gained [26]. Investments in advanced dustproof equipment would be a total 843 million RMB; the ratio of investment to restored economic losses was 1:1.43. Controlling workplace dust concentrations is critical to reducing the onset of pneumoconiosis and achieving economic benefits [26]. Therefore, strengthening management, implementation of effective wet work, adequate individual protective equipment and automation, and the closed production of dust are the main measures for decreasing the risk of CWP [27].

## 5. Conclusions

Based on survival analysis data, it is important to focus on CWP patients with complications—especially those with pulmonary tuberculosis—or earlier initial dust exposure year for improving their survival rate. The patients need more aggressive follow-up investigations to extend their life through preventing complications.

## Figures and Tables

**Table 1:** Cox regression variable assignment table.

Variable Name	Assignment Description
Initial dust exposure year (X1)	Continuous variable
Dust exposure years (X2)	Continuous variable
Age of onset (X3)	Continuous variable
Time since first exposure (X4)	Continuous variable
First diagnostic stage (X5)	Object of observation = 0, Stage I = 1, Stage II = 2, Stage III = 3
Work type (X6)	Mining = 1, Transportation assistance = 0
Complicated with pulmonary tuberculosis or not (X7)	Yes = 1, No = 0

**Table 2:** 495 cases of Coal Workers’ Pneumoconiosis (CWP) patients with cumulative survival rate and its standard error.

Age Groups	Observed Patients ^1^	Surviving Patients ^2^	Died Patients ^3^	Cumulatively Observed Patients ^4^	Corrected Observed Patients ^5^	Mortality Rate ^6^	Survival Rate ^7^	Cumulatively Survival Rate	Cumulatively Mortality Rate	Death Probability
25~	6	4	2	495	493	0.0041	0.9959	0.9959	0.0041	0.0201
30~	21	17	4	489	480.5	0.0083	0.9917	0.9877	0.0123	0.0408
35~	65	43	22	468	446.5	0.0493	0.9507	0.9390	0.0610	0.2193
40~	83	51	32	403	377.5	0.0848	0.9152	0.8594	0.1406	0.3497
45~	71	61	10	320	289.5	0.0345	0.9655	0.8297	0.1703	0.1590
50~	78	62	16	249	218	0.0734	0.9266	0.7688	0.2312	0.3101
55~	71	69	2	171	136.5	0.0147	0.9853	0.7575	0.2425	0.0707
60~	37	35	2	100	82.5	0.0242	0.9758	0.7392	0.2608	0.1143
65~	35	32	3	63	47	0.0638	0.9362	0.6920	0.3080	0.2752
70~	28	26	2	28	15	0.1333	0.8667	0.5997	0.4003	0.5000

^1^ CWP patients in this age group; ^2^ CWP patients that still alive; ^3^ died CWP patients; ^4^ the cumulative CWP patients in observation period; ^5^ the corrected CWP patients in observation period; ^6^ died patients/Corrected observed patients; ^7^ Mortality rate.

**Table 3:** Life expectancy of CWP patients.

Age Groups	Cumulatively Observed Patients	Deceased Patients	Mortality Rate	Death Probability	Surviving Patients	Projected Died Patients	Surviving Person Years	Total Surviving Person Years	Life Expectancy
25~	495	2	0.0041	0.0201	100,000	2008	494,980	2,562,275	25.6
30~	489	4	0.0083	0.0408	97,992	3996	479,971	2,067,295	21.1
35~	468	22	0.0493	0.2193	93,996	20,617	418,438	1,587,325	16.9
40~	403	32	0.0848	0.3497	73,379	25,663	302,739	1,168,886	15.9
45~	320	10	0.0345	0.1590	47,716	7586	219,617	866,148	18.2
50~	249	16	0.0734	0.3101	40,130	12,444	169,543	646,531	16.1
55~	171	2	0.0147	0.0707	27,687	1957	133,542	476,988	17.2
60~	100	2	0.0242	0.1143	25,730	2941	121,299	343,445	13.3
65~	63	3	0.0638	0.2752	22,790	6272	98,267	222,146	9.7
70~	28	2	0.1333	0.5000	16,517	8259	123,879	123,879	7.5

**Table 4:** Comparison of the mortality rate and life expectancy for subjects without pneumonia, pulmonary tuberculosis, lung cancer and pulmonary heart disease.

Age Groups	Cumulatively Observed Patients	Life Expectancy	Without the Death Cause of Pneumonia			Without the Death Cause of Pulmonary Tuberculosis			Without the Death Cause of Lung Cancer			Without the Death Cause of Pulmonary Heart Disease		
			Deceased Patients	Death Probability	Life Expectancy	Deceased Patients	Death Probability	Life Expectancy	Deceased Patients	Death Probability	Life Expectancy	Actually Died Patients	Death Probability	Life Expectancy
25~	495	25.6	2	0.0201	29.9	2	0.0201	27.0	2	0.0201	26.8	2	0.0201	27.0
30~	489	21.1	2	0.0206	25.4	4	0.0408	22.5	4	0.0408	22.3	4	0.0408	22.5
35~	468	16.9	14	0.1466	20.9	18	0.1839	18.4	21	0.2106	18.2	20	0.2018	18.3
40~	403	15.9	23	0.2671	19.1	28	0.3143	16.9	27	0.3051	17.3	27	0.3051	17.3
45~	320	18.2	4	0.0675	20.1	10	0.1590	18.6	8	0.1297	18.8	9	0.1445	18.8
50~	249	16.1	12	0.2439	16.4	14	0.2778	16.6	15	0.2941	16.3	14	0.2778	16.6
55~	171	17.2	2	0.0707	15.8	1	0.0361	17.0	2	0.0707	17.0	1	0.0361	17.0
60~	100	13.3	2	0.1143	11.9	2	0.1143	12.6	1	0.0592	13.1	2	0.1143	12.5
65~	63	9.7	2	0.1942	8.1	3	0.2752	8.9	3	0.2752	8.8	3	0.2752	8.8
70~	28	7.5	1	0.2941	4.4	2	0.5000	6.3	1	0.2941	6.2	2	0.5000	6.2

**Table 5:** Multivariate Cox regression analysis of 495 CWP patients.

Variable	Regression Coefficients	Standard Error	Wald χ^2^ Value	*p* Value	RR	95% CI
Initial dust exposure Age	−0.104	0.015	47.025	<0.001	0.902	0.875–0.929
Age of onset	−0.059	0.016	13.729	<0.001	0.943	0.914–0.973
First diagnosis of age	0.640	0.171	14.020	<0.001	1.897	1.357–2.653

RR: relative risk; CI: confidence interval.
